# DiscoverySpace: an interactive data analysis application

**DOI:** 10.1186/gb-2007-8-1-r6

**Published:** 2007-01-08

**Authors:** Neil Robertson, Mehrdad Oveisi-Fordorei, Scott D Zuyderduyn, Richard J Varhol, Christopher Fjell, Marco Marra, Steven Jones, Asim Siddiqui

**Affiliations:** 1 Canada's Michael Smith Genome Sciences Centre, British Columbia Cancer Research Centre (BCCRC), British Columbia Cancer Agency (BCCA), Vancouver, BC, Canada

## Abstract

DiscoverySpace is a graphical application for bioinformatics data analysis. Users can seamlessly traverse references between biological databases and draw together annotations in an intuitive tabular interface. Datasets can be compared using a suite of novel tools to aid in the identification of significant patterns. DiscoverySpace is of broad utility and its particular strength is in the analysis of serial analysis of gene expression (SAGE) data. The application is freely available online.

## Abstract 2

DiscoverySpace, a graphical application for bioinformatics data analysis, in particular analysis of SAGE data, is described

## Rationale

Underlying DiscoverySpace, the DiscoveryDB relational database integrates 26 biological databases (Table 1). Although relational databases are indispensable tools for large-scale data analysis, they present a technically challenging interface. DiscoverySpace provides user interfaces that help researchers to conceptualize, visualize and manipulate available datasets, allowing them to construct powerful queries without the requirement of programming knowledge and experience.

DiscoverySpace was developed to support serial analysis of gene expression (SAGE) [1] technologies, and throughout the paper we illustrate the features of the application with scenarios from example SAGE analyses. Other examples are provided to show how DiscoverySpace is applicable to a wider range of bioinformatics use cases.

The paper does not focus on the details of the low-level implementation, but instead describes the approach, the architecture of the application, conceptual underpinning and use of key technologies such as the Resource Description Framework (RDF) [2]. We introduce the various user interfaces of DiscoverySpace, explain the functionalities made available, and, where possible, contrast it with other available tools. We show that DiscoverySpace offers an innovative and extensible example of a graphical bioinformatics environment. The application and code are freely available to academic researchers.

## Biological database integration

Bioinformatics is a data-driven discipline in which the available data sources dictate the scope of possible research. Biological data are dynamic; new databases are constantly being created [3], and existing databases are constantly updated and extended. It remains a challenge to integrate the data and analyze them in an effective manner.

The problem of integrating biological databases is well known [4]. Our approach has been to centralize all data into a relational database where they can be shared and readily accessed. A drawback of this 'data warehousing' method is the ongoing need to maintain the database and develop data import tools [4]; though many groups, including this one, have successfully managed to sustain such an effort over time [5,6].

A key feature of the 'data warehousing' method is that it concentrates all of the data at a single physical location. This allows complex and highly optimized queries to be run at the site of data storage, with resulting gains in efficiency and performance. The alternative, a more distributed 'federated' solution, draws data from a number of remote servers before processing and returning the result [7,8]. Federated systems amalgamate content from multiple data warehouses, therefore permitting the organizational independence of each data provider. Distributed systems are still an emerging technology, with rapidly evolving standards and best practices [9]. We chose to concentrate our efforts on utilizing the capabilities of one database, leaving the challenge of supporting multiple databases to a later stage of development.

## The DiscoveryDB database

The DiscoveryDB database supports 26 biological databases, including Ensembl [10], Gene Ontology (GO) [11], Refseq [12], Entrez [13], Mammalian Gene Collection (MGC) [14] and Uniprot [15] (Table 1). The database also hosts data generated by the Genome Sciences Centre (GSC), such as the results of SAGE experiments.

At present, many biological data providers do not publish their data in a database-compatible tabular format, and require specialized analysis and parsing to prepare them for import into a relational database. Proprietary flat-file formats, such as those used by the Uniprot and GenBank [16] databases, centralize all of an entity's data into a single document-like record, and are well suited to access by UNIX command line tools and scripting languages. Unfortunately, such proprietary formats make efficient mass analysis using relational databases much more difficult. Recently, many data providers, such as Entrez, GO and Ensembl, have begun to publish data files in a tabular, tab-separated format. Such files are optimal because they can be directly imported into a database with little, or no, additional processing. Such files are also easily accessible via traditional UNIX tools.

The DiscoveryDB database is housed in a MySQL database server [17] (presently being upgraded to PostgreSQL [18]) that supplies all of the data content for the DiscoverySpace application. Because data sources are frequently updated, we have developed software to automatically download and import data files in a series of regular update cycles. Data files are parsed, if necessary, using dedicated parsing tools and then imported into the central database system.

## Accessing the data

Once the various data sources have been imported into DiscoveryDB's central relational database, researchers need a means to access the data. While SQL provides a powerful interface to the database, gaining full command of the SQL language can be challenging and time-consuming for those not trained as programmers.

The most rudimentary method to promote data access is to provide a list of documented, 'pre-canned' SQL queries; a researcher can adapt a query to suit their needs and then execute it in a script or database client. The GO database [11] provides such example queries. This solution does require a degree of technical confidence from the researcher, but requires little development. It has the disadvantage that the researcher needs to rework all their queries when the data structure changes.

An alternative is to develop tools that wrap the database query with another interface, such as a web interface or API (application programming interface). Web interfaces typically provide a form to capture parameters, and produce a chart or other report given those parameters; DAVID [19] and FatiGO [20] are examples of web interfaces. For the more programming-literate researcher, some biological databases provide APIs. These APIs wrap SQL calls in programming interfaces and save the researcher from having to analyze the data model and code the SQL themselves; the Ensembl database [10] and GO database [11] provide such APIs. APIs assume a level of comfort with the given programming language.

Most tools are narrowly focused and, depending upon the sophistication of the implementation, restrict the user to a finite number of specific questions: for instance, 'get the Refseq accessions for these GenBank accessions', or 'get the GO terms for these genes at level 4', and so on. In such instances the interface and underlying query are dedicated to one particular usage, so the researcher does not have free rein over the data but is restricted to those functionalities that the developer exposes. For more complex tasks the researcher will need to learn and integrate multiple interfaces into a single methodology.

Because of the dynamic nature of the available data, and because of the rapidity with which researchers alter their methodologies, it is a challenge for developers to keep tools current and relevant. This is particularly acute in the case of API development where multiple programming languages are supported, as is the case with the SeqHound [5] and Atlas [6] projects. The developer must struggle to anticipate future analyses, as well as maintain the existing functionality.

## Development strategy

The strategy of the DiscoverySpace project has been to develop a comprehensive graphical interface that supports all possible data models with only minimal configuration on the part of the database administrator. We have aimed to create an application that allows the researcher to explore the available knowledge domain freely with a limited amount of training, to expose the content and power of the underlying database while abstracting away its low-level complexity.

We decided to develop a graphical standalone application rather than a browser-based application. Standalone applications are more difficult to develop, but permit a richer user experience as there is more scope for customization. Standalone applications can also make full use of the features of the client computer, rather than offloading all work to the server (which is a shared resource). Throughout the application we have used familiar interactive devices that enhance user productivity, such as 'drag and drop' functionality. 'Drag and drop' is used to exchange data between DiscoverySpace's various internal tools; throughout the application it is possible to define a dataset in one tool, then drag it out and drop it onto another tool. We have also consistently provided features that promote interoperability with external applications, such as 'cut and paste'.

## The DiscoverySpace architecture

**Figure 1:**
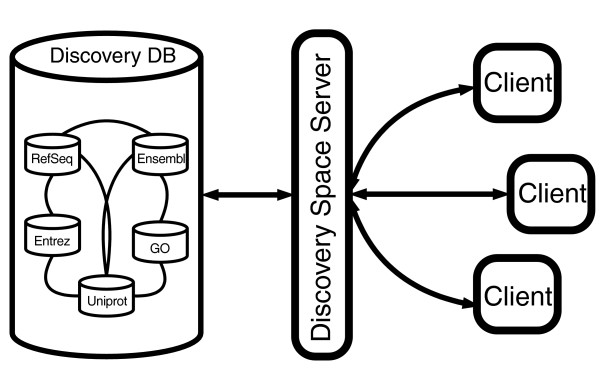
Diagram showing the three-tier architecture of DiscoverySpace. Many DiscoverySpace clients connect to the shared DiscoverySpace server using HTTP and DiscoverySpace's application-level protocol. Each DiscoverySpace server connects to a single database server using the database's JDBC (Java Database Connectivity) driver.

DiscoverySpace is a distributed application in which multiple DiscoverySpace clients connect to a single DiscoverySpace server. The application is built around the three-tier architecture widely used by distributed applications (Figure 1); with database, middleware and client components. The server-side middleware controls access to the database and provides additional application logic, while the client provides a feature-rich graphical user interface, storage and data processing.

Both client and server-side components are written in the Java programming language [21]. The main strengths of Java are that it is object-oriented, platform independent, and offers a wealth of well-designed APIs. The middleware component is a Java servlet [22] and is deployed in the Apache Tomcat [23] reference servlet container. The client is distributed using Java Web Start technology [24], which integrates with the user's desktop and updates the application automatically as newer versions are released.

The middleware layer decouples the client and the database so that database drivers do not need to be deployed with the standalone client; the underlying database implementation can be changed without needing to re-release the client software. This decoupling is particularly vital when considering that future versions of DiscoverySpace may progress to a federated architecture with many servers per client, each of which might use a database from a different vendor. Future versions would also benefit from a server discovery protocol that would enable the client to find and identify available DiscoverySpace servers.

As each DiscoverySpace client starts up, it contacts its configured server and retrieves a schema describing the available data content. The client then communicates with the server using DiscoverySpace's custom protocol to query and download data. The protocol, which uses RDF/XML [25] in the request and tab-separated data in the response, is designed and optimized specifically for DiscoverySpace interactions. Each request is authenticated using the user's name and password, and the server has the ability to restrict data types and to filter content based upon the user's permissions. This means that confidential or sensitive information can be limited to specific collaborators.

## The DiscoverySpace data model

A data model is an abstract framework for data representation that determines how data are conceptualized and understood. A data model acts as a common definition of terms for both the user and the developer, and needs to offer broad descriptive power and extensibility, while remaining simple and intuitive. Like the basic architecture, the data model is fundamental and determines the capabilities of the application; finding the correct model is vital.

Many groups have used ontologies, or controlled vocabularies, to describe biological knowledge domains: for example the GO [26] and Sequence Ontology [27] projects. Models with ontological support are advantageous because they help to describe the semantics of the data rather than merely the syntax. While SQL is extremely good at defining the format of data, it is poor at describing meaning. If data are properly annotated with rich ontological meta-information, in addition to their syntactic constraints, then they are truly self-describing.

Prototypes of DiscoverySpace used an ontological data model provided by the KDOM API [28]. However, in this latest iteration we have adopted the Jena API [29], which provides full support for the Resource Description Framework (RDF) [2] and its associated ontology languages (DAML+OIL [30], OWL [31]). RDF is a widely used metadata language and is the foundation of other bioinformatics projects such as BioMOBY [9]. By annotating relational data with RDF metadata, data integration occurs at the semantic level, not the syntactic level [32].

RDF conceptualizes data as graphs of atomic and compound nodes connected by edges known as predicates, or properties. RDF graphs are formally described using statement-like structures called triples, each of which comprises a subject, a predicate and an object. An example triple would be 'gene NM_032983 translates to protein NP_116765', where the gene and protein are subject and object, respectively, and "translates to" is the predicate. Compound nodes, termed resources, may be both the subject and object of a triple. Atomic nodes, or literals, can only be the object. RDF mandates that globally accessible resources should have a worldwide web-friendly universal resource identifier (URI). DiscoverySpace adopts a specialized form of URI designed for the biological knowledge domain: Life Science Identifiers [33].

**Figure 2:**
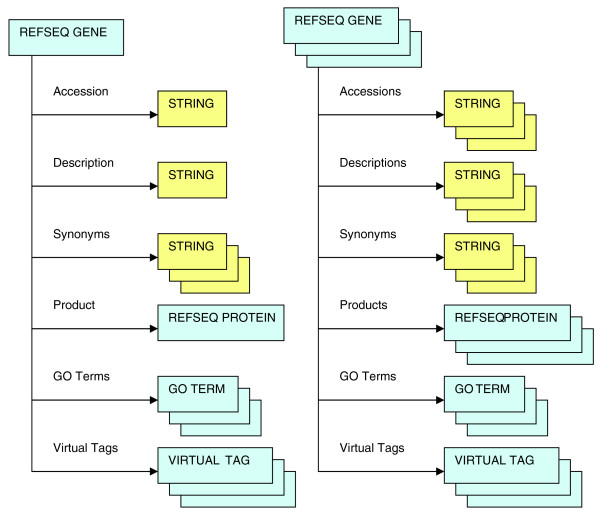
A diagram depicting two RDF graphs. The color yellow represents literal nodes and the color blue represents resource nodes. The capitalized text denotes the data type of each node. The arrows represent properties connecting the subject resource to object nodes, each with its own label. The left hand graph represents an individual RDF resource and its properties. Note that some properties have a single object whereas some have multiple objects. The right-hand graph represents a parallelization of the left-hand graph. Instead of a single subject node it has a root set of subject nodes, and properties follow to the objects of all subjects. Notice that the properties that were singular in the left-hand graph are now plural, and have multiple objects.

While it is possible to deal with only individual resources and their individual properties, the DiscoverySpace model also parallelizes the RDF model into sets of subject resources, their properties and the grouped sets of object resources (Figure 2). For instance, as a gene resource 'translates to' a protein resource, so a set of genes 'translates to' a set of proteins. The DiscoverySpace model is thus conceptualized as a tree of typed sets linked by properties, cascading down from a root subject set of resources. That root dataset might be imported from an external source or defined internally using a query.

## Supporting SAGE analysis

The features of DiscoverySpace are illustrated through SAGE analysis use cases; therefore, it is necessary to introduce the pertinent aspects of a SAGE experiment. SAGE is a gene expression profiling technology [1]. The result of a SAGE experiment is a library of SAGE tags, in which a tag is derived from a transcribed RNA sequence. A tag has a quality score (derived from PHRED [34] values) and a sequence, ten or more base pairs in length (depending upon the protocol used), that can be used to identify the corresponding transcript. SAGE libraries can be compared to other libraries to identify common or differential patterns of expression. A typical SAGE analysis scenario is composed of three stages: first, specify tag sequences; second, compare tag sequences and perform statistical analysis; and third, map tag sequences to genes and proteins for interpretation.

This specific use case can be extended to a general bioinformatics scenario: importing and defining datasets; performing quantitative and qualitative analysis on given datasets; and mapping data to available annotations for semantic interpretation.

The capabilities of DiscoverySpace will be illustrated by two example experiments. These examples provide a biological context to showcase the features of the application and its underlying database.

### Example one

In the first example, we compare the expression of two sets of short SAGE tags: one a set of tags from a library generated from a normal pancreas tissue, the other the combined set of tags from two pancreatic cancer libraries. The sets are compared using the Audic-Claverie [35] significance test and those sequences that are significantly up- and down-regulated (to 95% confidence) are isolated. The isolated sequences are then mapped to Refseq transcripts, via position one, sense strand virtual tags. The functional qualities of the Refseq transcripts are analyzed using GO annotations. Functions of particular interest are reviewed and interpreted by the researcher; those genes that are associated with significant functions are then selected and mapped back to the dataset of up- and down-regulated tag sequences.

### Example two

In the second example, we compare five Cancer Genome Anatomy Project (CGAP) breast long SAGE libraries; four from cancer samples and one from normal tissue. Logical analysis is performed to isolate those non-singleton tag sequences that are present in all of the cancer libraries and not at all in the normal library. Those isolated sequences are then mapped to their counterpart virtual tags, to Refseq transcripts, to their Entrez genes and to predicted subcellular localizations generated from the translations of the transcripts (using PSORT [36]). With this additional annotation the researcher can identify genes of further interest, for example, those that are predicted to be extracellular. These tag sequences are then compared with other available long SAGE libraries to determine whether the tags are significantly expressed in comparison to a broader range of samples.

## Importing and defining datasets

**Figure 3:**
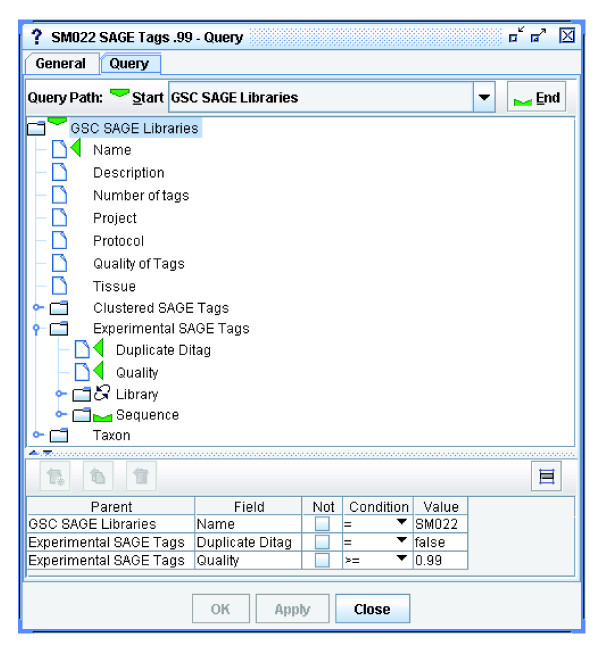
A screenshot of the DiscoverySpace query 'SM022 SAGE Tags .99'. The SAGE data housed by the Discovery database are represented with three classes of resource: SAGE Libraries, SAGE Tags and Tag Sequences. A library represents an experiment performed on a tissue sample; a library has properties such as a name and a protocol and is composed of many thousands of SAGE tags. Each SAGE tag represents a discrete, physical result from a SAGE experiment, and has a quality score, a read identifier, in addition to ditag and linker flags. Each tag also has a tag sequence that represents the sequence of the tag, such as TTCATACACCTATCCCC. In this figure, the user is requesting those tags from the library SM022 that have a quality score ≥0.99 and were not extracted from duplicate ditags.

SAGE tag data can be imported into DiscoverySpace either from tag-frequency files or directly from raw fasta files. The data may be used immediately or saved for later use. The import includes PHRED [34] sequence quality scores, if they are available. In addition to data loaded by the user, the DiscoverySpace database houses over 300 publicly available SAGE libraries published by the Gene Expression Omnibus (GEO) [37] and the CGAP [38]. Once the data have been imported into DiscoverySpace, the user can specify the libraries they wish to analyze (Figure 3).

## Performing quantitative and qualitative analysis on given datasets

DiscoverySpace integrates commonly used tools for performing statistical analysis of SAGE data. Specifically, these tools are the Scatterplot and Venn table.

**Figure 4:**
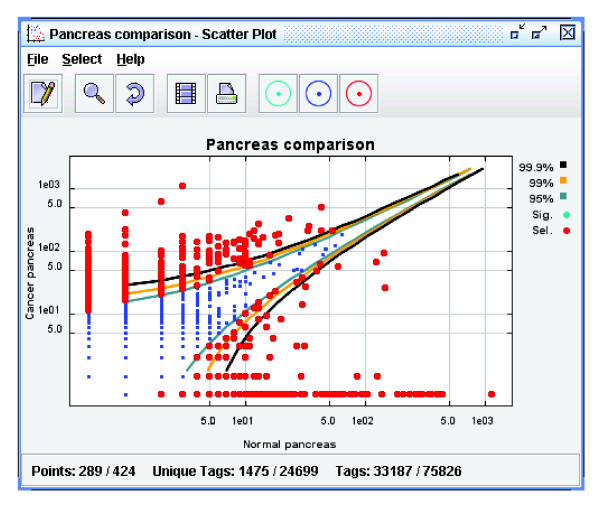
A screenshot of the DiscoverySpace Scatterplot. To use the Scatterplot the user must define a comparison between two sets of tag sequences. For this example the researcher has constructed a comparison of one normal pancreas library on the x-axis versus two cancer libraries on the y-axis. This comparison has then been viewed in the Scatterplot and the researcher has selected those tags that are up- and down-regulated with a confidence threshold of 95% or greater (marked in red). Selected tags can be dragged out of the chart and isolated into their own dataset 'Up & down regulated pancreas' for further investigation.

The Scatterplot (Figure 4) implements the Audic-Claverie significance test [35] to plot a chart that visualizes similarly and differentially expressed sequences. The Audic-Claverie method, which accounts for different sample sizes, was designed for the quantitative, absolute comparison of SAGE gene expression profiles. Although we chose the Audic-Claverie method for our initial implementation, other methods for evaluating differentially expressed tags have been developed. Chen *et al*. [39] have developed a Bayesian method for assigning *p *values to differentially expressed genes and this is available through SAGE Genie [40]. Vencio *et al*. [41] have also developed a Bayesian method that is available through WebSAGE [42].

Data points on the Scatterplot chart can be selected manually or by setting criteria of up- or down-regulated confidence thresholds. Points can also be selected by dropping tag sequences from outside the Scatterplot onto the chart; this allows the user to visualize the relative expression of a given set of tags with regards to the comparison. The tags represented by the selected data points can be dragged out of the chart for further analysis using other DiscoverySpace tools.

**Figure 5:**
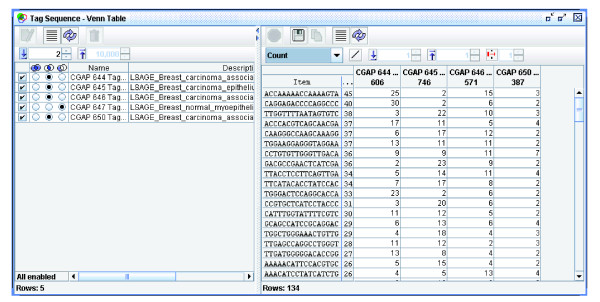
A screenshot of the DiscoverySpace Venn table. In the example above the user has specified five sets of tag sequences from CGAP SAGE libraries and has selected and dragged them into the Venn table. Four of the sets are tag sequences from breast cancer libraries, the fifth, CGAP 647, is from a normal breast sample. The user has raised the quantitative cutoff to 2 or above in order to exclude singleton tag sequences, and has then excluded any tags in the normal set and has selected the intersection of the other cancer sets. The resulting sets of tags are selected from the table and are dragged out for further analysis in the DiscoverySpace Explorer.

The Venn table (Figure 5) allows the user to perform set manipulations and statistical analysis upon multiple sets of data resources. In the first stage of Venn analysis the user can apply a quantitative filter across the contents of each imported set. For example, this allows the user to exclude genes with low expression values. In the second stage, the user can apply a logical filter that performs set operations upon imported datasets. In the third stage the user applies a statistical view to the resulting sets to compare and contrast the contents. And in the fourth and final stage another quantitative filter can be applied to further restrict the statistical view.

## Mapping data to available annotations for semantic interpretation

**Figure 6:**
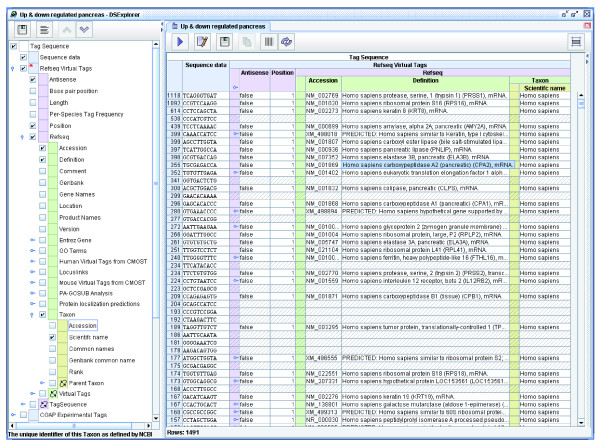
A screenshot of the DiscoverySpace Explorer. The Explorer comprises one 'view', which describes the cross-section of data required by the user, and one or more datasets, which provide the initial starting content. The view is displayed in the left panel of the Explorer and graphically represents all navigable paths as a tree of sets cascading from the root class (much like the data model from Figure 2). Some properties have a literal object, such as a name, a sequence or a comment field. Others are links to associated resources such as genes, proteins or pathways. The view determines the content being displayed in the main table of the Explorer; each property in the tree has a checkbox that is used to include or exclude the property as a column. The right panel of the Explorer holds the main display. Each dataset added to the Explorer is represented by a tab containing a table. Each table displays the member resources of the selected dataset (and their weight values) as rows. The properties of the resources, and the properties of associated resources, are represented as columns, as determined by the view. The table, with its novel nested header, reflects the structure and the color-coding of the view in the left panel. If no view is specified by the user then a default view is created from the class of the dataset. All datasets in an Explorer session must have the same data type, and that class must be shared by the root node of the view. In the example above the user has constructed a view consisting of a path from the root set of tag sequences 'Up & down regulated pancreas' through virtual tags from Refseq to their counterpart Refseq genes. Additionally, the user has restricted the set of linked virtual tags to only those from human Refseq (multi-species joins are supported by the data model) and only those tags at position one (closest to the 3' end) on the sense strand. The result of the mapping operation, a set of human Refseq genes, can be selected and dragged out of the Explorer for further interpretation. The frequency, or weight, is displayed on the left hand side of each tag sequence.

The Explorer is DiscoverySpace's central data exploration and visualization tool (Figure 6). The Explorer allows the user to map from a set of data resources to directly and indirectly associated resources. The tool attempts to mask the complexity of the underlying database joins and queries behind an intuitive, but powerful, spreadsheet-like interface. In database terms, the Explorer performs a series of outer joins, in contrast to the Query tool, which performs a single inner join.

As with the query, the Explorer allows the user to attach constraints to the view to filter any associated sets. This can help to reduce datasets to an informative and manageable amount. For example, a constraint can reduce the set of all associated Refseq genes to only those associated Refseq genes that are human, non-predicted and located on chromosome 1. Constraints can be attached to any non-literal node.

Data in the Explorer can be manipulated in many ways, including tag to gene mapping, and assignment of annotations (for example, GO terms, PSORT annotations) to genes.

## Tag to gene mapping with the CMOST database

Several quality resources exist to assist investigators in tag assignment, notably the NCBI SAGEmap [43] and SAGE Genie [40] efforts. These resources focus primarily on identifying genes that, in general, have been highly characterized or have significant expressed sequence tag (EST) data. SAGE Genie uses multiple (seven) ranked transcript sources to map tags to genes focusing on the more abundant tags and ignoring tags with single base variations with respect to the reference sequence or tags that occur only once. SAGEmap also provides mappings to ESTs. For both SAGEmap and SAGE Genie, mappings are predefined by an algorithm.

We have implemented a database that allows the user to choose the data source to which tags are mapped. They may choose to map (concurrently) to one or more of RefSeq [12], MGC [44] and Ensembl [45] genes. They may also map tags directly to the genome. The results of the mapping are presented in the DiscoverySpace Explorer.

**Figure 7:**
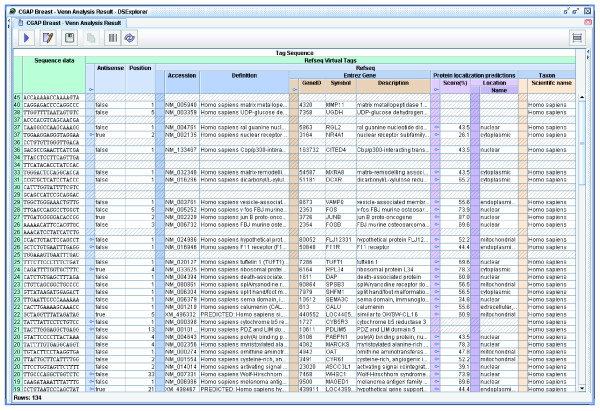
A detail from the main table of the DiscoverySpace Explorer showing the ability to draw together multiple annotations. The user has taken the resulting tags from the Venn analysis and is viewing them in the DiscoverySpace Explorer. The user has mapped the tags to their human Refseq genes, via virtual tags. The user is also viewing various qualities of those Refseq genes, their Entrez gene counterparts and predicted subcellular locations (generated using PSORT [36]). Hatched cells indicate the absence of a mapping.

Mappings are performed against a set of pre-extracted tags. For RefSeq, MGC and Ensembl genes, the tag adjacent to every *Nla*III site (sense and antisense) in the gene is extracted (10 base pairs for SAGE tags and 17 base pairs for LongSAGE tags). For mapped tags, the DiscoverySpace Explorer displays both the sense of the tag relative to the gene and the ordinal count of the *Nla*III site relative to the 3' end of the gene. In Figure 7, the columns indicate whether the tag is antisense relative to the gene and the position or ordinal rank of the *Nla*III site. The first tag maps to position 1 or the 3' most *Nla*III site in the gene, while the second maps to position 6 or the 6th *Nla*III site relative to the end of the gene. For the genome, tags adjacent to all *Nla*III sites are extracted and the DiscoverySpace Explorer reports the position and strand of the mapped tags.

A unique feature of the application is that it allows the user to map 'off-by-one' tags. During the construction of and sequencing of SAGE libraries, single base pair errors (insertions, deletions and permutations) may be incorporated into tag sequences to create off-by-one tags. Several groups have developed methods to cluster off-by-one tags with the highly expressed tag from which they are derived [46-49]. Imperfect tag clustering and the presence of a single nucleotide polymorphism in the tag sequence for the individual gene under study means that some high frequency off-by-one tags will not be mapped by standard methods.

The comprehensive mapping of SAGE tags (CMOST) database allows the user to map tags to RefSeq, MGC and ENSEMBL genes and to the genome, allowing for the possibility of single base pair insertions, deletions and permutations in tag sequences. This is achieved by pre-populating the CMOST database with the off-by-one mapped location of all experimentally observed tags. All possible one-off tags are generated for each experimental observed tag. Those off-by-one sequences that match an exact map to a sequence database (the same set of pre-extracted tags described previously) are stored in the database for later retrieval. As new SAGE libraries are sequenced and additional tag sequences generated, the off-by-one calculations are performed for new tags.

The user may elect to utilize the off-by-one mappings or not and has complete control over the entire tag mapping process.

The tag clustering and off-by-one mapping features are only available for LongSAGE libraries (comprising 21 base pair tags). Tags from regular SAGE libraries (14 base pair tags) are too short and map to too many locations for these features to be effective.

## Drawing together multiple annotations with the DiscoverySpace Explorer

The DiscoverySpace Explorer enables the researcher to navigate and view multiple annotation paths at once, so that it is possible, for instance, to view both associated Refseq genes and associated MGC genes, and even the proteins of those genes, concurrently in the same table (Figure 7).

**Figure 8:**
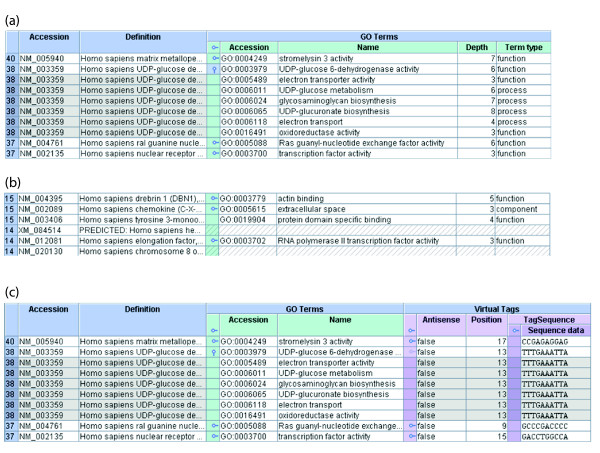
Displaying multiple annotations through expansion points. **(a) **A detail from the Explorer showing an open expansion point. The blue outer columns represent a set of Refseq genes, the green inner columns represent the GO terms mapped to those genes. As there are many GO annotations per gene, the property is represented by an expansion point. The second expansion point is expanded and the others collapsed. One can see that the expansion is represented by the product of the subject and objects of that property. The repeated subject cells are shaded for clarity. Expansion points in the header allow each property to be expanded or collapsed in bulk per column. **(b) **A detail from the Explorer showing various expansion point states. Notice that the third Refseq gene does not have an expansion point because it has only one GO annotation. The fourth and sixth genes have no GO annotations at all, as represented by the hatched cells. The first, second and fifth genes have multiple GO annotations, all collapsed. **(c) **A detail from the Explorer showing conflicting expansion points. The user is now viewing the set of Refseq genes, their GO annotations and their virtual tags (lilac), along with the sequences of those virtual tags (purple). Properties 'GO Terms' and 'Virtual Tags' are sibling one-to-many properties of the class Refseq gene and are, therefore, 'in competition'. The user has expanded the GO annotations of the second Refseq gene and the product is being displayed. Notice that the respective 'expansion point' for the virtual tags property is now dimmed and the first virtual tag is now repeated as part of the GO product. If the dimmed 'expansion point' is expanded, then the open competing property (GO Terms) will automatically be closed.

A strict tabular format is necessary for easy compatibility with other tools such as Microsoft Excel, and all data from the Explorer are exportable as tab-separated value (TSV) files. However, a relationship may be one-to-many (a subject can have many objects of a particular property): for example, a gene can have many GO terms, or many synonyms. One-to-one properties are simple to display in a tabular format because all qualities of a resource can be represented on a single row. However, it is more difficult to display one-to-many relationships where, to stay tabular, it is necessary to show the product of the subject and objects of a property, and repeat the subject for each object. The Explorer makes the relationships clear by shading out repeated subjects, and their properties, which are the result of such products (Figure 8).

The representation of one-to-many properties is complicated by the fact that sibling, one-to-many properties are 'in competition'. The product of a gene and its synonyms is simple to comprehend because it reflects the hierarchy of the model and the path from gene to synonym. However, the product of a gene's synonyms and the gene's GO terms is slightly obscure and does not reflect a path in the hierarchy. The Explorer protects the user against such situations by dimming expansion points if they are in conflict with already open expansion points (Figure 8). Simultaneous expansions are only possible if the properties are nested and the expansions follow exactly one path down the hierarchy. If a subject resource has an expanded one-to-many property then that property will be collapsed if a competing property is expanded.

## Conclusion

DiscoverySpace is a supportable and extensible software application; the architecture is strong and scaleable, and the core functionality has wide utility. The application allows a user to traverse multiple biological databases without requiring detailed knowledge of the source databases and provides useful domain-specific tools. The application presents a consistent, uniform view of the data, simplifying the process of analysis.

Further development will include adding further client-side logic and visualizations for domain-specific functionalities. Effort is also required to complete the DiscoverySpace server and release it as a standalone distribution. This will entail upgrading the client application for multi-server support and polymorphic queries.

A particular aim is to strengthen DiscoverySpace for development by third-parties. Though we are not yet at the stage of having a stable and publishable API, DiscoverySpace has a well-defined internal structure and strong feature set. Continuing work will develop the core application into a general bioinformatics platform. The application and code are freely available at [50].

## Figures and Tables

**Table 1:** Discovery data sources and their update frequency

Data source	Update frequency (days)*	Present in
CGAP (SAGE) [38]	60	DiscoveryDB/DiscoverySpace
COG [51]	60	DiscoveryDB/DiscoverySpace
Ensembl (human and mouse) [45]	30	DiscoveryDB/DiscoverySpace
EntrezGene [13]	14	DiscoveryDB/DiscoverySpace
Gene Expression Omnibus (SAGE) [37]	60	DiscoveryDB/DiscoverySpace
Gene Ontology [11]	30	DiscoveryDB/DiscoverySpace
Homologene [52]	30	DiscoveryDB/DiscoverySpace
Inparanoid [53]	30	DiscoveryDB/DiscoverySpace
KEGG [54]	60	DiscoveryDB/DiscoverySpace
LocusLink [55]	21	DiscoveryDB/DiscoverySpace
MGC [44]	14	DiscoveryDB/DiscoverySpace
PAGOSUB [56]	60	DiscoveryDB/DiscoverySpace
PFAM [57]	30	DiscoveryDB/DiscoverySpace
PSORT [36]	120	DiscoveryDB/DiscoverySpace
RefSeq [12]	14	DiscoveryDB/DiscoverySpace
SwissProt [58]	90	DiscoveryDB/DiscoverySpace
Taxonomy (NCBI) [52]	90	DiscoveryDB/DiscoverySpace
TCAG [59]	30	DiscoveryDB/DiscoverySpace
Transcompel* [60]	-	DiscoveryDB/DiscoverySpace
Transpro* [61]	-	DiscoveryDB/DiscoverySpace
Hugo [62]	120	DiscoveryDB only
Omim [63]	60	DiscoveryDB only
Genecards [64]	When released	DiscoveryDB only
Trembl [58]	90	DiscoveryDB only
Interpro [65]	120	DiscoveryDB only
SCOP [66]	120	DiscoveryDB only

Many data sources are not released publicly to coincide with a consistent release cycle and, as such, an automated pipeline has been created to regularly monitor the release of new data. Data sources present in DiscoveryDB have been integrated and can be accessed via SQL commands. Data sources present in DiscoverySpace can, in addition, be accessed through the DiscoverySpace graphical user interface. *Licensed data sources (not externally available).
